# A primer of bone metastases management in breast cancer patients

**DOI:** 10.3747/co.2008.176

**Published:** 2008-01

**Authors:** B. Petrut, M. Trinkaus, C. Simmons, M. Clemons

**Keywords:** Bone metastases, breast cancer, skeletal-related events, bisphosphonate

## Abstract

Bone is the most common site for distant spread of breast cancer. Following a diagnosis of metastatic bone disease, patients can suffer from significant morbidity because of pain and skeletal related events (sres). Bisphosphonates are potent inhibitors of osteoclastic function and the mainstay of bone-directed therapy for bone metastases. The aims of bisphosphonates are to prevent and delay sres, to reduce bone pain, and to improve quality of life. Bisphosphonate therapy appears to have revolutionized treatment of bone metastases, but bisphosphonate use has several limitations. Those limitations include the high cost of the agents and the need for return trips to the clinic for intravenous treatment. Moreover, many uncertainties surround bisphosphonate use—for example, the timing of bisphosphonate initiation, the choice of bisphosphonate to use, the optimal duration of treatment, and the appropriate means to identify patients who will and will not benefit. In addition, potentially serious adverse effects have been associated with bisphosphonate use—for example, renal toxicity, gastrointestinal side effects, and osteonecrosis of the jaw. The present review is intended as a primer for oncology specialists who treat patients with bone metastases secondary to breast cancer. It focuses on bisphosphonate treatment guidelines, the evidence for those guidelines, and a discussion of new therapeutic agents. It also discusses the use of biochemical markers of bone metabolism, which show promise for predicting the risk of a patient’s developing a sre and of benefiting from bisphosphonate treatment.

## 1. INTRODUCTION

Bone is the most common site of breast cancer recurrence 1,2. Despite modern cancer therapy, up to two thirds of patients with bone metastasis will subsequently develop an skeletal-related event (sre), defined as any of pathologic fracture, a requirement for surgical intervention and palliative radiotherapy to bone lesions, hypercalcemia of malignancy, and spinal cord compression. Not only are sres associated with significant morbidity, they also negatively affect survival. Moreover, sres are associated with loss of mobility and social functioning, and reduction in quality of life (qol)^2^.

Treatment of bone metastases ideally involves a multidisciplinary team, including medical oncologists, radiation oncologists, palliative care specialists, and orthopedic surgeons. Systemic treatment aimed at delaying the progression of bone metastases may include endocrine therapy, biologic agents, chemotherapy, and oral or intravenous bisphosphonate therapy. New osteoclast inhibitors are currently under investigation and may offer alternative treatment options for these patients in the future.

## 2. BISPHOSPHONATES

Bisphosphonates are an established standard of care for patients with bone metastases. Table I reviews the American Society of Clinical Oncology (asco) and Cancer Care Ontario guidelines regarding bisphosphonate use in breast cancer patients with bone metastases 3,4. Bisphosphonates are potent inhibitors of osteoclast-mediated bone resorption through several mechanisms, including induction of osteoclast apoptosis, inhibition of osteoclast maturation and differentiation, and reduced osteoclast activity 5. In addition, bisphosphonates may act directly on tumour cells by inducing apoptosis, inhibiting matrix metalloproteinase 1, inhibiting angiogenesis, decreasing adhesion of tumour cells within bone, and reducing levels of vascular endothelial growth factor 6.

### 2.1 Bisphosphonate Trials and Meta-analyses

The clinical benefits of bisphosphonate therapy in secondary prophylaxis (that is, their use in patients with breast cancer and established bone metastases) have been demonstrated in a large number of placebo-controlled trials and meta-analyses 7–12. As compared with placebo, zoledronic acid, pamidronate, clodronate, and ibandronate have all been shown to reduce the risk of sres and to increase time to first sre^8^–^19^ (Table II).

A large meta-analysis encompassing eighteen studies (five of which were conducted in breast cancer patients) indicated that, as compared with placebo, bisphosphonates significantly reduced the odds ratios (ors) for non-vertebral fractures [or: 0.80; 95% confidence interval (ci): 0.64 to 0.99], combined fractures (or: 0.75; 95% ci: 0.61 to 0.93), need for radiotherapy (or: 0.65; 95% ci: 0.54 to 0.79), need for orthopedic surgery (or: 0.59; 95% ci: 0.43 to 0.83), and hypercalcemia (or: 0.43; 95% ci: 0.29 to 0.63), but not for spinal cord compression 20. Similarly, in a Cochrane systemic review that included twenty-one randomised studies involving bisphosphonate use among breast cancer patients 21, nine of those studies demonstrated a risk reduction (rr) of 17% (rr: 0.83; 95% ci: 0.78 to 0.89; *p* < 0.00001) for developing a sre with bisphosphonate use. Intravenous zoledronic acid 4 mg was most effective in reducing the risk of developing a sre by 41% (rr: 0.59; 95% ci: 0.42 to 0.82). In seven of the studies, bisphosphonates, when compared with placebo, significantly improved bone pain 21.

The benefits of bisphosphonate therapy for various sres seem to be time-dependent; that is, the bisphosphonate must be given for at least 6 months before an effect is seen on skeletal morbidity outcomes and for at least 12 months before a reduction in the need for orthopedic surgery becomes significant 20. To date, treatment with bisphosphonate does not appear to improve overall survival 21.

Results from clinical trials evaluating the analgesic properties of bisphosphonates vary considerably, mostly because of differences in the patient populations and pain assessment scales 22,23. Placebo-controlled trials of intravenous pamidronate, ibandronate, and zoledronate have demonstrated improvement in pain scores 7,12,22. Two published comparative trials have reported an advantage for intravenous pamidronate over oral clodronate in reducing metastatic bone pain 24. A systemic review by Wong and Wiffen concluded that although evidence supported bisphosphonate effectiveness in providing some pain relief for bone metastasis, the data were insufficient to recommend bisphosphonates as first-line therapy for metastasis-related bone pain 25. New strategies of bisphosphonate administration for bone pain are currently being explored 26.

Bisphosphonates are remarkably variable in structure and potency. The newer, nitrogen-containing bisphosphonates such as ibandronate, pamidronate, and zoledronic acid are several orders of magnitude more potent than earlier-generation bisphosphonates such as clodronate 27. Comparative bisphosphonate trials have attempted to ascertain the superiority of one bisphosphonate regimen over another, with endpoints being reduction in the incidence of sres and improvement of pain and qol (Table III). One study showed that pamidronate was superior to clodronate with regard to pain (*p* < 0.01) and improvement in biochemical markers of bone turnover 24. Another trial demonstrated the superiority of zoledronic acid (4 mg intravenously over 15 minutes) over pamidronate (90 mg intravenously over 2 hours); patients treated with zoledronic acid had an increased time to first sre (*p* = 0.013) and fewer sres (*p* = 0.58) 29. In that study, the proportion of patients with at least 1 sre was similar for zoledronic acid and pamidronate, but zoledronic acid reduced by 20% the overall risk of any skeletal complications developing (rr: 0.799; *p* = 0.025)^29^. When phase iii trial data are compared, ibandronate and zoledronic acid seem to have similar efficacy 30. That hypothesis is currently being tested in two large randomized phase iii trials (Table IV) 31,32.

### 2.2 Uncertainties About Bisphosphonate Use in Clinical Practice

The evidence of clinical benefit from bisphosphonates in breast cancer is overwhelming. Attention is now concentrated on defining the optimal time to start treatment, duration of treatment, and importantly, predicting which patients are most likely to benefit from either repeat bisphosphonate administration or a switch to an alternative bisphosphonate 33 (Table V).

### 2.3 Which Patients Benefit Most from Bisphosphonate Use?

Randomized controlled trials have shown that the beneficial effects of bisphosphonates are time-dependent; significant benefits were seen only after 6 months of treatment 20. Therefore, looking at survival times for patients with breast cancer is important before starting them on bisphosphonate treatment.

A retrospective analysis involving 859 patients who developed bone metastases from breast cancer showed that, as compared with patients with bone and visceral metastases, patients with disease confined to the skeleton were most likely to develop a sre^34^ . The difference with regard to the development of a sre was probably attributable to the survival difference between the groups (median survival for patients with bone-only disease was 2.2 years as compared with 5.5 months for patients with bone and liver metastases). Patients with bone-only disease may therefore benefit most from treatment with bisphosphonates, because they are most likely to live long enough to experience the time-dependent benefits of bisphosphonates 34. Notably, Canadian treatment guidelines do not encourage the use of bisphosphonate treatment in patients with a life expectancy below 6 months and who are asymptomatic from their bone metastases 3.

Along the same lines, most bisphosphonate trials have enrolled a disproportionate number of patients with bone-only disease who, as a consequence of their prolonged survival, are at greater risk of developing sres than are patients with (for example) visceral disease. Therefore, the magnitude of the benefit seen in bisphosphonate trials probably exceeds the benefit seen in clinical practice. Tables II and III provide an overview of placebo-controlled trials and comparative trials of bisphosphonate use in patients with bone metastases.

Further prospective studies are needed to identify the subgroup of patients most likely to develop sres and therefore to benefit most from bisphosphonate treatment. In an attempt to develop a prediction model, our group is currently analyzing baseline clinical characteristics in 100 patients on bisphosphonates at three cancer centers 35.

### 2.4 When Should Bisphosphonates Be Started in Patients with Newly Diagnosed Bone Metastases?

There is a paucity of data for optimal use of bisphosphonates, mainly in regard to initiation and treatment duration. According to asco guidelines, bisphosphonate therapy should be started in patients with metastatic breast cancer who have imaging evidence of lytic bone destruction. Furthermore, asco considers it “reasonable” to start intravenous bisphosphonates in breast cancer patients with an abnormal bone scan despite normal plain radiographs, provided that the patients are reporting concordant localized pain. Intravenous bisphosphonate treatment is not recommended for asymptomatic patients with abnormal bone scans whose plain radiographs are normal 4. Canadian guidelines do not restrict the indications for bisphosphonate therapy to patients with lytic bone destruction on imaging 3. Despite these recommendations, most trials suggest that, given the potential of bisphosphonates to delay time to first sre, bisphosphonates should be started when bone metastases are diagnosed, even when patients are asymptomatic 21,27.

## 3. CHOOSING A BISPHOSPHONATE

Current Cancer Care Ontario guidelines advocate starting patients with newly diagnosed bone metastases on intravenous pamidronate or oral clodronate as first-line treatment. In other North American centers and in Europe, most patients with breast cancer metastatic to bone are started on zoledronic acid as first-line treatment 36.

Oral clodronate is approved for patients with breast cancer, and it should be considered for patients who cannot attend frequent clinic appointments or who choose to decline intravenous therapy. The efficacy of clodronate in preventing skeletal morbidity has been shown in placebo–controlled clinical trials of women with lytic bone disease (Table ) II 8–10. However, in clinical practice, the potential for adverse gastrointestinal effects and the very low absorption rates (<5%) of oral clodronate even under ideal conditions may contribute to poorer outcomes and poor patient compliance 27.

Comparative trials have shown the superiority of pamidronate over clodronate for pain control, and the superiority of zoledronic acid over pamidronate for delaying the first sre and reducing the incidence of sres (Table III). Ongoing comparative studies are trying to clarify which bisphosphonate is best to use in clinical practice (Table IV). Interestingly, in one study, patients receiving oral ibandronate after prior intravenous pamidronate significantly preferred the oral regimen 30.

## 4. OPTIMAL DURATION OF BISPHOSPHONATE TREATMENT

The asco guidelines recommend that bisphosphonate treatment continue until there is evidence of a substantial decline in a patient’s general performance status (Table I). A recent Canadian study confirmed that 90% of patients continue bisphosphonate treatment until death, despite repeated sres and bone progression 37. Criteria to guide response to bisphosphonate therapy and the optimal and appropriate duration of bisphosphonate administration are lacking. The decision to continue, stop, or switch to an alternative bisphosphonate remains empirical and based on personal experience. Studies have shown that patients with skeletal disease progression and pain despite the use of oral clodronate or intravenous pamidronate may achieve an improvement in pain control and a reduction in levels of bone turnover markers after switching from pamidronate to zoledronic acid or to ibandronate 30,38. Our group is starting a phase iii trial to define the role of, and best time to switch patients to, a more potent bisphosphonate treatment after either bone disease progression or development of a sre while on first-line bisphosphonate treatment 39.

Unfortunately, despite treatment with even the most potent bisphosphonate (zoledronic acid), one third of patients will develop further sres within 2 years of initiating therapy. In addition, bisphosphonate side effects such as renal toxicity, nausea, vomiting, flu-like symptoms, and osteonecrosis of the jaw are becoming increasingly prominent concerns as the use of these agents continues to increase 40,41. Furthermore, bisphosphonates are expensive agents; they have a substantial impact on the oncology drug budget. In a *post hoc* economic assessment of two multinational trials, the cost of pamidronate was projected to greatly exceed the cost savings associated with preventing a sre^40^. However, a cost–utility analysis performed in Canada of prophylactic pamidronate for prevention of sres suggested that pamidronate offered breast cancer patients with bone metastases a substantial quality–adjusted benefit at a reasonable cost 42.

The existing cost-effectiveness data are difficult to apply across different health care systems 43. Importantly, maintaining patients on bisphosphonate treatment indefinitely has major financial implications with unknown benefits. Pharmacoeconomic evaluations should be therefore combined with clinical trials to accurately predict the true cost of this supportive treatment and, ultimately, to assess the optimal use of these agents.

## 5. MARKERS OF BONE RESORPTION

In recent years, advances in understanding the mechanism of bone metastases have led to the discovery of several potential markers for dysregulation of bone coupling. The most widely used markers are urinary N-terminal crosslinked type 1 collagen telopeptide (ntx) and C-terminal cross-linked type 1 telopeptide. Urinary ntx 44 levels have been shown 45–47 to predict

- occurrence of sres in breast cancer patients with bone metastases,
- response to bisphosphonates,
- pain scores, and
- patient outcomes.

Several studies have shown a strong correlation between moderate (50–100 nmol/mmol creatinine) and high (≥ 100 nmol/mmol creatinine) levels of ntx and the number of sres or deaths (or both) in patients with bone metastasis 44,48.

Furthermore, ntx seem to be valuable in assessing how pain responds to bisphosphonates. In the pooled analysis of phase iii trials of zoledronic acid, significant reductions in ntx were accompanied by significant declines in bone-pain scores and lesser increases in analgesic use at 96 weeks 48. Our own group conducted a study with breast cancer patients that showed a reduction in ntx levels after patients on a second-generation bisphosphonate were switched to a third-generation bisphosphonate after development of a sre or progressive bone disease. The decline in ntx levels was an important predictor for palliative pain response to both ibandronate 30 and zoledronic acid 38.

Overall, the hope is that biochemical markers will serve as adequate surrogates for further assessment of bisphosphonate efficacy. However, given the lack of sufficient, rigorous phase iii data, current asco guidelines advise against the use of biochemical markers to monitor bisphosphonate treatment routinely 4. The role of bone markers in guiding bisphosphonate treatment is currently being tested in a large National Cancer Research Institute–supported phase iii clinical trial in the United Kingdom (bismark, *n* = 1400). In that trial, patients with breast cancer–associated bone metastases are being treated with zoledronic acid, either on a regular schedule of 4 mg intravenously every 3–4 weeks, or as indicated by ntx levels. The primary endpoint is development of a sre. Secondary endpoints include qol, pain, analgesic use, health economics, change in systemic therapy, and survival.

## 6. NEW AGENTS TARGETING THE MECHANISM OF BONE METASTASES

In metastatic bone disease, an imbalance occurs between the action of osteoblasts and that of osteoclasts, with net bone loss resulting. A triad of molecules has been shown to regulate the maturation, differentiation, and survival of osteoclasts:

- receptor activator of nuclear factor κB (rank),
- rank ligand (rankl), and
- osteoprotegerin (opg) 49.

The rankl/opg ratio is significantly increased in patients with severe osteolytic bone metastases. Therefore, targeting the rank–rankl–opg pathway is a promising intervention for treating metastatic bone disease, particularly among patients who are refractory to potent bisphosphonates 49.

Denosumab (amg 162) is a human monoclonal antibody that binds to and inhibits rankl with high affinity and specificity, mimicking the effect of endogenous opg. A recent phase ii study of patients with bone metastases showed that, as compared with pamidronate, denosumab is significantly more likely to suppress urinary ntx. That study concluded that the dose of 120 mg every 4 weeks is optimal for future trials 50,51. Another randomized study of 255 bisphosphonate-naïve breast cancer patients with bone metastasis found denosumab to be at least as effective as intravenous bisphosphonates in reducing the risk of sres. A phase iii trial comparing denosumab with zoledronic acid is ongoing in patients with bone metastases 51,52.

## 7. SUMMARY

Many systemic therapeutic options are available for patients with bone metastases, with none being completely satisfactory. Bisphosphonates have become the mainstay of practice, and their use in breast cancer patients with bone metastases is associated with a significant reduction and delay in sres and a reduction in bone pain. Despite the rapid integration of bisphosphonates into standard clinical practice, many uncertainties remain regarding their use: for example, the optimal bisphosphonate agent and duration of therapy, the most beneficial scheduling regimen, and identification of the patients most likely to benefit from bisphosphonate treatment.

Currently, Cancer Care Ontario guidelines advocate for the use of intravenous pamidronate or oral clodronate for patients with metastatic bone disease secondary to breast cancer. Zoledronic acid is largely reserved for clinical trials or for patients who are intolerant to intravenous pamidronate. Meanwhile, elsewhere in North America and Europe, zoledronic acid is first-line therapy for metastatic bone disease among breast cancer patients.

Further research is merited to identify factors that accurately predict the subgroups of patients at highest risk for developing bone metastases and subsequent complications and the patients that will benefit most from bisphosphonate treatment. Markers of bone turnover seem to hold the most promise for identifying patients likely to benefit from bisphosphonate treatment and for guiding the decision to discontinue bisphosphonate therapy or to switch to an alternative bisphosphonate. Finally, new osteoclast inhibitors are currently under investigation, and these agents may offer effective treatment options with reduced toxicity for patients with bone metastases.

## Figures and Tables

**TABLE I:** American Society of Clinical Oncology (asco) and Cancer Care Ontario (cco) guidelines for bisphosphonate (bp) use in bone-metastatic disease in breast cancer patients 3,4

	*ASCO (2003 update)*	*CCO (2004 update)*
Recommended bp	Intravenous pamidronate or zoledronic acid. Evidence is insufficient to support the efficacy of one bisphosphonate over the other.	Oral clodronate, intravenous pamidronate, or zoledronic acid.
Initiation of bp for prevention of skeletal-related events	Reasonable to consider BP treatment in women with normal plain radiographs who demonstrate bone destruction in other imaging. Starting BPs in women with only an abnormal bone scan but without evidence of bone destruction is not recommended.	Recommendations for bps are not restricted to patients with osteolytic metastases. All women with breast cancer who have bone metastases should be offered bps. An exception should be patients with a short expected survival (that is, less than 6 months), who have well-controlled bone pain.
Role in pain management	The presence or absence of bone pain should not be a factor in initiating bps.	In patients with bone metastases and pain, treatment with pamidronate, zoledronate, or clodronate may be a useful adjunct to conventional measures for pain control.
Discontinuation	Treatment with bps to be continued until evidence appears of substantial decline in the patient’s performance status.	No evidence from clinical trials addresses the optimal duration of bp use.

**TABLE II:** Overview of placebo-controlled trials of bisphosphonates in advanced breast cancer

*Trial*	*Bisphosphonate*	*Dose*	*Results*
Paterson *et al.,* 1993 9	Clodronate	Oral, 1600 mg daily	Reduced the event rate of vertebral fractures and deformity, and the combined event rate for all events.
Kristensen *et al.,* 1999 8	Clodronate	Oral, 400 mg twice daily	Reduced the number and significantly delayed the time to first SRE.
Tubiana–Hulin *et al.,* 2001 10	Clodronate	Oral, 1600 mg daily	Significantly delayed the time to first bone event and significantly reduced pain intensity and analgesic use.
Hortobagyi *et al.,* 1998^11^	Pamidronate	Intravenous, 90 mg every 3–4 weeks	Reduced the incidence and delayed the onset of SREs.
Theriault *et al.,* 1999^13^	Pamidronate	Intravenous, 90 mg every 4 weeks	Reduced skeletal morbidity and the incidence of SREs and delayed the onset of SREs.
Lipton *et al.,* 2000 14	Pamidronate	Intravenous, 90 mg every 3–4 weeks	Reduction in the percentage of patients with >1 SRE, median time to first SRE extended by nearly 6 months, and reduction in the mean skeletal morbidity rate was found.
Hultborn *et al.,* 1999^15^	Pamidronate	Intravenous, 60 mg every 4 weeks	Significantly fewer SREs
Conte *et al.,* 1996 16	Pamidronate	Intravenous, 45 mg every 3 weeks	Effective in delaying the time to progression of bone lesions.
Body *et al.,* 2003^17^	Ibandronate	Intravenous, 2 or 6 mg every 3–4 weeks	Significantly reduced the smpr by 20% and extended the time to first SRE.
Body *et al.,* 2004^18^	Ibandronate	Orally, 50 mg daily	Significantly reduced the smpr as compared with placebo in a combined analysis.
Tripathy *et al.,* 2004^19^	Ibandronate	Orally, 20 mg or 50 mg daily	Significantly reduced the smpr as compared with placebo.
Kohno *et al.,* 2005^12^	Zoledronic acid	Intravenous, 4 mg every 4 weeks	Significant multiple event analysis demonstrated a 44% reduction in the risk of developing a SRE.

sre = skeletal-related event; smpr = skeletal morbidity period rate.

**TABLE III:** Overview of completed comparative trials of bisphosphonates in bone metastases

*Trial*	*Patients*	*Bisphosphonate*	*Primary outcome*	*Conclusions*
Jagdev *et al.,* 2001 24	*n=*51 [various primary cancers (22 breast)]	Clodronate: Oral, 1600 mg daily (group 1) Intravenous, 1500 mg loading, then oral 1600 mg daily (group 2) Pamidronate: Intravenous, 90 mg every 3 weeks (group 3)	Use pain scores and ntx to compare efficacy of two schedules of clodronate with intravenous pamidronate	Pamidronate was more effective than clodronate with regard to pain control (*p<*0.01). No statistically significant difference in NTX evident between groups.
Rosen *et al.,* 2002^28^	*n=*1648 (myeloma and breast cancer)	Zoledronic acid: Intravenous infusion, 4 mg or 8 mg over 15 minutes Pamidronate: Intravenous infusion, 90 mg over 2 hours every 3–4 weeks	Use skeletal-related events (sres) and pain score to compare efficacy of zoledronic acid with that of pamidronate	In subgroup of breast cancer patients (*n=*1130), zoledronic acid had significant clinical benefit as compared with pamidronate: prolonged time to first sre, 310 days vs. 174 days, *p=*0.013; and reduced incidence of SREs, mean 1.2 vs. 2.4 events per year, *p=*0.008.

ntx = N-terminal crosslinked type 1 collagen telopeptide.

**TABLE IV:** Overview of ongoing comparative trials of bisphosphonates in metastatic breast cancer

*Trial*	*Patients (*n*)*	*Bisphosphonate*	*Duration of study*	*Primary outcome*	*Secondary outcomes*
Southwest Oncology Group S0308 31					
	488	Ibandronate: oral, 50 mg daily vs. Zoledronic acid: intravenous, 4 mg every 4 weeks	18 Months	New skeletal-related event (sre)	Time to first sre, quality of life, overall survival, safety
Zoledronate versus Ibandronate Comparative Evaluation 32					
	1400	Ibandronate: oral, 50 mg daily vs. Zoledronic acid: intravenous, 4 mg every 4 weeks	96 Weeks of treatment with follow-up for further 3 years	Multiple event analysis: sres over 96 weeks	Proportion of patients experiencing new SRE, time to first event, quality of life, safety

**TABLE V:** Summary of bisphosphonate use for metastatic bone disease in breast cancer patients

Placebo-controlled trials in breast cancer patients with bone metastases confirm significant reductions in the incidence and delay in the occurrence of skeletal-related events (SREs) with bisphosphonate use.
Effects of bisphosphonates are time-dependent; in terms of reducing SREs, benefits begin to be identified after 6 months of treatment.
The benefits of bisphosphonate treatment in patients with poor prognosis are mostly unknown.
Which bisphosphonate to use as first-line therapy remains to be clarified. Evidence mainly supports the use of intravenous aminobisphosphonates. However, clodronate can be offered to patients who are unable or unwilling to come to hospital for intravenous treatment.
The absolute magnitude of bisphosphonate benefit and the who, when, and how long parameters of treatment remain unclear.
A switch to a more potent bisphosphonate (zoledronic acid or ibandronate) after either a SRE or bone metastasis progression during treatment with a first-line bisphosphonate (clodronate or pamidronate) may offer better pain control.
